# Huangqi Guizhi Wuwu Decoction Ameliorates Cachexia in Lung Cancer Mice Through Regulating Amino Acid Metabolism: Insights From Transcriptomics, Metabolomics and Experimental Validation

**DOI:** 10.1111/jcmm.71387

**Published:** 2026-09-27

**Authors:** Yingjia Zhou, Shaoli Zhao, Hongmei Shen, Jia Wu, Mei Li, Shan Liu, Jian Yang, Xiaowei Jin, Fan Li, Huantian Cui

**Affiliations:** ^1^ The Third Affiliated Hospital of Kunming Medical University, Yunnan Cancer Hospital, Peking University Cancer Hospital Yunnan Integrated Traditional Chinese and Western Medicine Department Kunming China; ^2^ First Teaching Hospital of Tianjin University of Traditional Chinese Medicine Tianjin China; ^3^ School of Pharmacy Kunming Medical University Kunming China; ^4^ Science and Technology Achievement Transformation Center of Kunming Medical University Kunming China; ^5^ First School of Clinical Medicine Yunnan University of Chinese Medicine Kunming China

**Keywords:** amino acid metabolism, cachexia, Huangqi Guizhi Wuwu Decoction, transcriptomics, untargeted metabolomics

## Abstract

The effects and mechanisms of Huangqi Guizhi Wuwu Decoction (HGWD) in cancer cachexia remain unclear. We generated an in vivo cachexia model by implanting Lewis lung carcinoma cells into mice and treated animals with HGWD. We evaluated muscle function using grip strength and wire‐grip tests. We also assayed markers of muscle synthesis and oxidative stress, and assessed tumour growth to evaluate any antitumor effects of HGWD. Finally, we performed transcriptomic and untargeted metabolomic analyses. HGWD increased muscle strength and restored muscle mass in cachectic mice. HGWD improved levels of proteins associated with muscle anabolism and altered oxidative stress–related factors. Treated mice showed modest tumour inhibition and greater food and water intake. Transcriptomic profiling of gastrocnemius muscle revealed significant enrichment of amino acid–related metabolic pathways after HGWD treatment. Untargeted metabolomics indicated that most branched‐chain amino acids were upregulated following HGWD intervention. Further validation showed that HGWD increased both gene and protein expression of adenosylmethionine decarboxylase 1 (AMD1) and spermine oxidase (SMOX), while decreasing gene and protein expression of glycine N‐methyltransferase (GNMT) in the gastrocnemius of cachectic mice. The anti‐cancer cachexia effect of HGWD appears to be associated with promoting the conversion of S‐adenosylmethionine (SAM) to spermidine, thereby correcting amino acid metabolic disturbances.

## 1 Introduction

Lung cancer remains one of the most common malignant tumours, with non‐small cell lung cancer (NSCLC) representing more than 85% of cases. Recent advances in immunotherapy and other treatment strategies have achieved significant clinical benefits in the first‐line management of advanced NSCLC, thereby improving patient outcomes [1]. Despite these advances, over 50% of patients with advanced NSCLC develop cachexia, which contributes to poor prognosis and reduced survival rates [2]. Cachexia is a multifactorial syndrome that arises primarily in the advanced stages of malignancy and is driven by systemic inflammation and profound metabolic dysregulation. It manifests as anorexia, progressive weight loss and skeletal muscle wasting, and it cannot be reversed with standard nutritional support. This syndrome markedly diminishes quality of life, impairs the efficacy of anti‐tumour therapies and shortens survival [3]. At present, no effective pharmacological treatments exist for tumour‐induced cachexia. Clinical management therefore relies on comprehensive multimodal strategies that target multiple pathways. These approaches aim to reduce inflammation, increase body weight—particularly lean body mass—enhance appetite and physical strength, mitigate fatigue and ultimately improve quality of life [4].

Skeletal muscle wasting is one of the most prominent and defining features of tumour‐induced cachexia. Nearly all patients with advanced solid tumours develop progressive loss of skeletal muscle mass and function at varying stages of disease, which substantially increases the risk of complications and mortality [5]. Amino acid metabolism plays a central role in sustaining muscle growth and homeostasis by supplying substrates for protein synthesis and regulating anabolic signalling pathways. This regulation is essential for preserving skeletal muscle mass and function [6]. Disturbances in amino acid metabolism accelerate muscle wasting in tumour‐induced cachexia through multiple mechanisms, whereas supplementation with essential amino acids, such as methionine, can partially slow disease progression [7]. Consequently, amino acid supplementation has become a cornerstone of therapeutic strategies in sports nutrition, age‐related sarcopenia and cancer‐associated cachexia [8, 9, 10].

Traditional Chinese medicine (TCM) formulas have long been applied as complementary and alternative therapies for cachexia, targeting multiple pathological processes, including systemic inflammation, skeletal muscle loss and metabolic dysregulation. For example, Fuzheng Xiaoai Decoction alleviates muscle wasting and renal atrophy while promoting weight gain in mouse models of tumour‐induced cachexia [11]. Similarly, Baoyuan Jiedu Decoction enhances mitochondrial function and thereby mitigates muscle atrophy in cachexia [12]. A retrospective clinical study of Shashen Maidong Decoction demonstrated its ability to relieve patient symptoms, improve nutritional status and reduce systemic inflammatory responses [13]. Systematic evaluation of the efficacy and mechanisms of TCM formulas in treating tumour‐induced cachexia may provide individualized, cost‐effective and well‐tolerated long‐term therapeutic strategies for patients with cancer.

Huangqi Guizhi Wuwu Decoction (HGWD) is a classical TCM formula with well‐documented immunomodulatory, anti‐inflammatory and microcirculatory regulatory properties [14]. Clinically, HGWD is widely used to treat conditions associated with metabolic dysfunction and immune dysregulation, particularly chemotherapy‐related side effects [15, 16]. Despite these applications, its therapeutic role and mechanisms in tumour‐induced cachexia remain poorly understood. In this study, we established an in vivo lung cancer–induced cachexia model by implanting Lewis lung carcinoma (LLC) cells into mice and treated them with HGWD to evaluate its efficacy in alleviating cachexia. We then performed transcriptomic and untargeted metabolomic analyses of the gastrocnemius muscle to investigate the molecular mechanisms underlying the observed effects. To our knowledge, this is the first study to examine HGWD in the context of lung cancer–associated cachexia. Our findings provide preliminary insights into its biological mechanisms and offer an experimental foundation for further development and clinical application of HGWD in the treatment of cancer cachexia.

## 2 Methods

### 2.1 Cells, Animals, Drugs and Reagents

LLC cells were purchased from Procell Life Science & Technology Co. Ltd. Eight‐week‐old specific pathogen‐free (SPF) male C57BL/6 mice (22–24 g) were obtained from Sibeifu Biotechnique Co. Ltd. Mice were housed in groups of five per cage under SPF conditions (temperature 22°C–25°C, relative humidity 50%–60%, 12 h light/12 h dark cycle) with ad libitum access to food and water. All animal procedures were approved by the Ethics Review Committee for Animal Experiments of Kunming Medical University (Ethics Approval Number: kmmu20241208).

All medicinal herbs required for preparation of HGWD were purchased from Kunming Medical University Campus Hospital. The formula was decocted in water and concentrated to the working dosage used in this study. Detailed preparation protocols are provided in the Supporting Information. We used UPLC‐Q‐TOF‐MS to qualitatively identify the chemical components of HGWD. Detailed analytical procedures and component information are provided in the Supporting Information. The information regarding other reagents and consumables used in the experiments is also listed in the Supporting Information.

### 2.2 Establishing an LLC‐Induced in Vivo Tumour Cachexia Model and Drug Intervention

After 1 week of acclimatization, we randomly assigned 50 C57BL/6 mice into five groups: a tumour‐free control group (Control), an LLC‐induced tumour cachexia model group (LLC), a cisplatin‐treated group (CDDP) and low‐dose and high‐dose HGWD treatment groups (L‐HGWD and H‐HGWD). Following the established experimental model for tumour‐induced cachexia reported in the literature [17], we generated the cachexia model by subcutaneously injecting an LLC cell suspension (approximately 1 × 10^6^ cells per mouse) into the right axillary region.

One week after inoculation, mice in the CDDP group received intraperitoneal injections of cisplatin (2 mg/kg) every other day. Mice in the L‐HGWD and H‐HGWD groups received daily gastric gavage of 3.3 and 13.2 g crude drug/kg/d of HGWD, respectively. Whereas the Control and LLC groups were administered an equal volume of saline by gavage for 14 consecutive days [18, 19, 20]. Throughout the study, mice had unrestricted access to food and water. We recorded body weight, food intake and water intake daily and measured tumour volume every 3 days. Behavioural assessments were conducted during the drug intervention period.

At the end of the 14‐day treatment, all mice were euthanized. We measured tumour weights and calculated the tumour inhibition rate. We also collected gastrocnemius, quadriceps and epididymal fat tissues, along with blood and gastrocnemius muscle samples for subsequent analyses.

Tumour volume was calculated using the formula:

$$V=a^{2}\times b\times 0.5$$

where *a* represents the short diameter (mm) and *b* represents the long diameter (mm) of the tumour.

Tumour inhibition rate was calculated using the formula:

$$N=\left(W_{1}-W_{2}\right)/W_{1}\times 100\%$$

where *W*_1_ represents the mean tumour weight (g) in the LLC group and *W*_2_ represents the mean tumour weight (g) in the treatment groups.

### 2.3 Behavioural Tests

We assessed neuromuscular function using the grip strength test (GST) and the wire grip test (WGT) following previously published protocols [18]. For GST, we used a commercial digital grip strength metre fitted with a metal grid to measure muscle strength on Days 0, 7 and 14. For the WGT (Day 14), each mouse was allowed to grasp a horizontal metal wire and we recorded the time until release. Each mouse underwent three tests.

### 2.4 HE Staining

We fixed harvested gastrocnemius muscles in 4% paraformaldehyde, dehydrated them through a graded ethanol series, cleared them in xylene and embedded them in paraffin. We cut 4‐μm sections, deparaffinized and rehydrated the slides and stained them with haematoxylin and eosin according to the HE staining kit manufacturer's instructions. Finally, we mounted the sections and imaged them by light microscopy.

### 2.5 Biochemical Assays

We collected gastrocnemius muscles and prepared tissue homogenates. We determined protein concentrations using the bicinchoninic acid (BCA) assay. As in previous studies [21, 22], following the manufacturers' instructions for the respective kits, we measured tissue levels of muscle glycogen, lactic acid, superoxide dismutase (SOD), glutathione peroxidase (GSH‐PX) and malondialdehyde (MDA).

### 2.6 Western Blot

We collected gastrocnemius muscles and prepared tissue homogenates for protein analysis. Protein concentrations were determined using the BCA assay. Equal amounts of protein samples were separated by SDS–PAGE and transferred onto polyvinylidene difluoride (PVDF) membranes. We blocked the membranes with 5% skim milk solution for 1 h at room temperature and then incubated them with the appropriate primary antibodies at 4°C overnight. After washing, we incubated the membranes with horseradish peroxidase–conjugated secondary antibodies for 2 h at room temperature. Protein bands were visualized using enhanced chemiluminescence (ECL) and quantified with ImageJ software. Protein expression was expressed as the ratio of the target protein band intensity to that of the internal reference band.

### 2.7 Transcriptomics

Transcriptome sequencing was carried out by Novogene Co. Ltd. (Beijing). The workflow included extraction of total RNA, assessment of RNA quality, purification of mRNA, fragmentation of mRNA, synthesis of complementary DNA (cDNA), enrichment of library fragments by PCR, quality control of sequencing libraries, high‐throughput sequencing using the Illumina platform and differential gene analysis. Differentially expressed genes (DEGs) were defined by the thresholds |Log_2_FC| ≥ 1 and *p* < 0.05. We performed KEGG pathway enrichment analysis using the clusterProfiler v4.0 package.

### 2.8 Untargeted Metabolomics

Untargeted metabolomics was conducted by Novogene Co. Ltd. (Beijing). Briefly, gastrocnemius muscles were collected, and metabolites were extracted using a methanol–acetonitrile–water system. Detection was carried out on a UHPLC‐Q Exactive HF‐X platform [23]. Raw data were processed, and metabolites were identified and quantified with Compound Discoverer 3.3 by comparison with the NovoMetDB‐UM‐V2.0 in‐house library [24]. Principal component analysis (PCA) and partial least squares discriminant analysis (PLS‐DA) were used for multivariate statistics. Differential metabolites were defined by VIP > 1, fold change (FC) > 1.5 or < 0.67 and *p* < 0.05. KEGG pathway enrichment was performed, with *p* < 0.05 considered significant.

### 2.9 Statistical Analysis

We performed statistical analysis using SPSS 22.0 software. Data are expressed as mean ± standard deviation. Multiple group comparisons were analysed by one‐way analysis of variance (ANOVA). When variances were homogeneous, the least significant difference (LSD) test was applied; when variances were heterogeneous, Tamhane's T2 test was used. Comparisons between two groups were conducted using independent samples *t*‐tests. Statistical significance was set at *p* < 0.05.

## 3 Results

### 3.1 Identification of the Main Components of HGWD Using UPLC‐Q‐TOF‐MS

We conducted qualitative profiling of the chemical constituents present in HGWD and HGWD‐containing medicated serum using UPLC‐Q‐TOF‐MS. Base peak chromatogram (BPC) acquired in both positive and negative ionization modes was shown in Figure 1. Among these identified constituents, 7 compounds, including 6‐Gingerol, Calycosin, Astragaloside IV, Paeoniflorin, Ethyl‐p‐methoxycinnamate, Oleanolic acid and Acetylacteoside, were confirmed as prototype compounds directly originating from HGWD and entering the systemic circulation (Tables S1 and S2).

**FIGURE 1:**
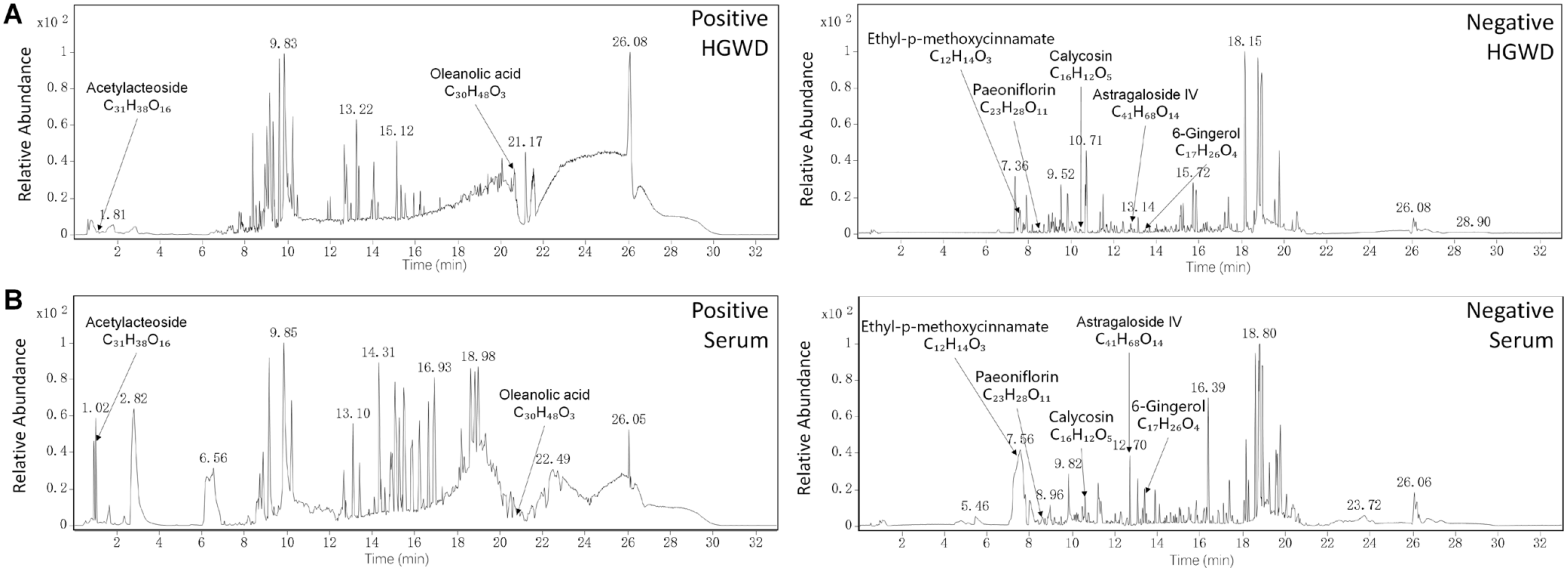
UPLC‐Q‐TOF‐MS analysis of HGWD extraction and serum samples. (A) Base peak chromatogram of HGWD in positive mode and negative mode. (B) Base peak chromatogram of HGWD‐containing serum in positive mode and negative mode.

### 3.2 HGWD Improves Lung Cancer Cachexia

After establishing the LLC‐induced tumour cachexia model in vivo, we first examined the effects of HGWD on muscle strength in cachectic mice. GST data showed no significant differences among groups at baseline (Day 0). By Days 7 and 14, mice in the LLC group exhibited a marked decline in muscle strength. Muscle strength in the CDDP group was comparable to or even lower than that of the LLC group. The L‐HGWD group showed no significant difference from the LLC group, whereas the H‐HGWD group demonstrated higher muscle strength than the LLC group (Figure 2A–C). The WGT produced similar results (Figure 2D), supporting the conclusion that HGWD improves muscle strength in cachectic mice.

**FIGURE 2:**
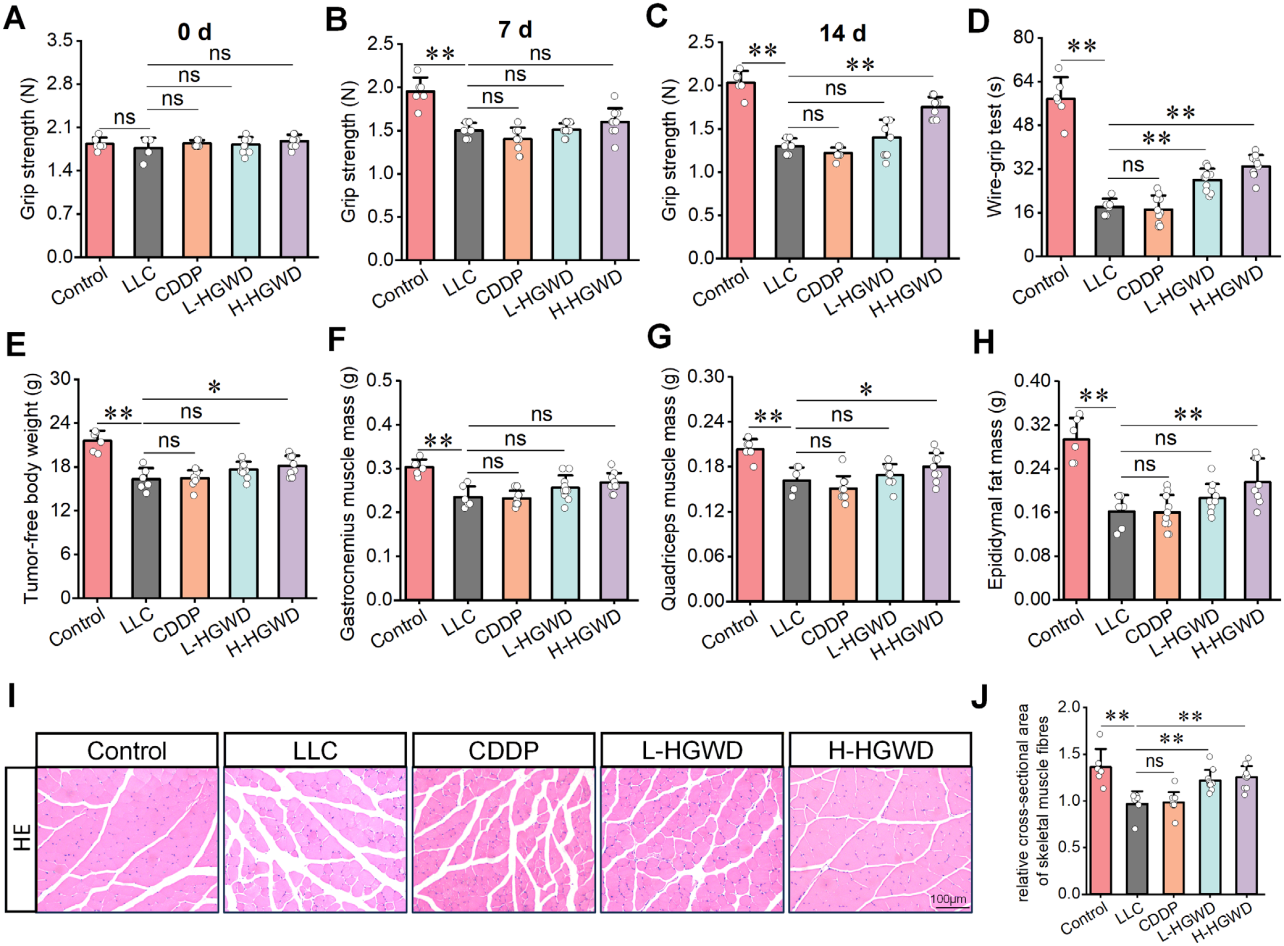
HGWD improves muscle strength and typical indicators in cachectic mice. HGWD enhances GST (A–C) and WGT (D) in cachectic mice, increases tumour‐free body weight (E), gastrocnemius muscle mass (F), quadriceps muscle mass (G) and epididymal fat mass (H) and augments skeletal muscle cross‐sectional areas (I, J). ns, Not significant, *p < 0.05, **p < 0.01.

We next assessed tumour‐free body weight, skeletal muscle mass and epididymal fat mass. Tumour‐free body weight, gastrocnemius muscle mass, quadriceps muscle mass and epididymal fat mass were all significantly reduced in the LLC group compared with the Control group. CDDP treatment failed to improve these parameters and, in some cases, further exacerbated them. In contrast, HGWD treatment improved body weight, muscle mass and fat mass to varying degrees (Figure 2E–H), suggesting that HGWD mitigates cachexia‐associated wasting.

Histological analysis further confirmed these findings. HE staining revealed a pronounced reduction in the cross‐sectional area of skeletal muscle fibres in the LLC group compared with the Control group. This reduction was aggravated by CDDP treatment but was attenuated by HGWD administration (Figure 2I,J), indicating that HGWD protects against muscle atrophy in cachectic mice.

We next examined proteins central to myogenesis in the gastrocnemius. Compared with the Control group, mice in the LLC group showed marked reductions in MYHC, MYOD1 and MYOG protein levels and a marked increase in the atrophy marker ATROGIN‐1. CDDP treatment did not significantly restore these myogenic proteins, whereas HGWD partially recovered their expression to varying extents (Figure 3A–E).

**FIGURE 3:**
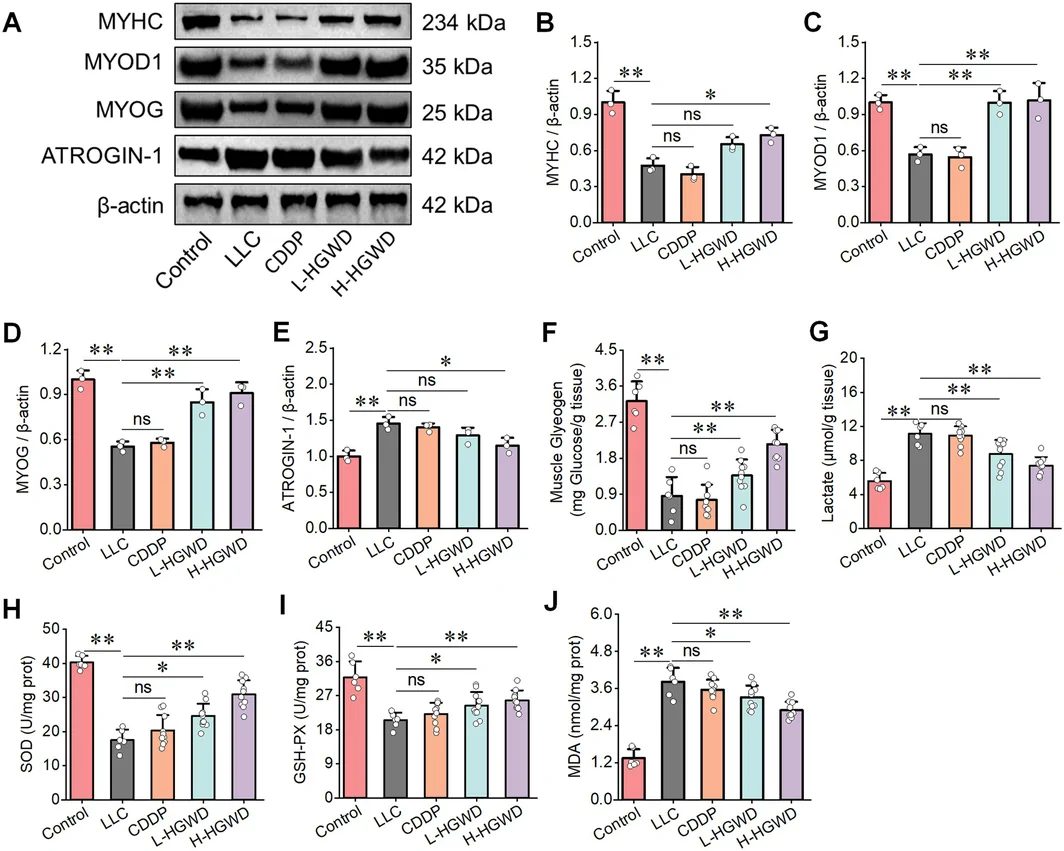
HGWD improves myogenesis and oxidative stress in cachectic mice. HGWD upregulates protein expression of MYHC, MYOD1 and MYOG (A–D) and downregulates protein expression of ATROGIN‐1 (A, E) in the gastrocnemius muscle of cachectic mice. It also elevates muscle glycogen levels (F), reduces lactate levels (G), increases SOD and GSH‐PX levels (H, I) and decreases MDA levels (J).

We then assessed muscle metabolic and oxidative‐stress parameters. In the gastrocnemius of LLC mice, muscle glycogen fell and lactic acid rose; antioxidant enzymes SOD and GSH‐PX declined while lipid peroxidation product MDA increased. CDDP failed to correct these abnormalities and in some measures exacerbated them; by contrast, HGWD ameliorated glycogen depletion, reduced lactic acid accumulation, increased SOD and GSH‐PX activities and lowered MDA levels to varying degrees (Figure 3F–J).

We also evaluated HGWD's effects on tumour burden. Relative to the LLC group, HGWD reduced both tumour volume and tumour weight, and the high‐dose HGWD regimen produced a clear tumour‐inhibitory effect (Figure S1A–C). Notably, mice receiving CDDP exhibited food and water intake comparable to or lower than LLC mice, whereas HGWD improved food and water consumption in cachectic animals (Figure S1D,E). Together, the above results suggest that HGWD has the potential to improve lung cancer cachexia.

### 3.3 Effects of HGWD on Cachectic Mice Transcriptome

We investigated the mechanisms by which HGWD alleviates lung cancer cachexia by performing transcriptome sequencing on gastrocnemius muscles from the Control LLC and H‐HGWD groups. We identified DEGs using |Log_2_FC| ≥ 1 and *p* < 0.05 as thresholds (Figure 4A,B), and then conducted KEGG pathway enrichment analysis. In the LLC vs. Control comparison, DEGs were significantly enriched in ‘Biosynthesis of amino acids’, ‘Cysteine and methionine metabolism’, ‘Arginine and proline metabolism’, ‘Cytokine–cytokine receptor interaction’, ‘Motor proteins’, ‘Glycolysis/Gluconeogenesis’, ‘Regulation of actin cytoskeleton’, ‘HIF‐1 signalling pathway’, ‘Pyruvate metabolism’ and ‘Carbon metabolism’. For the H‐HGWD vs. LLC comparison, DEGs were significantly enriched in ‘Arginine and proline metabolism’, ‘Biosynthesis of amino acids’, ‘Cysteine and methionine metabolism’, ‘Motor proteins’, ‘Galactose metabolism’, ‘PPAR signalling pathway’, ‘Cholesterol metabolism’, ‘Calcium signalling pathway’, ‘Thiamine metabolism’ and ‘Glycine, serine and threonine metabolism’. Most of these pathways are involved in metabolism, particularly amino acid metabolism (Figure 4C,D). Importantly, HGWD intervention markedly upregulated multiple genes related to ‘Motor proteins’, including Myhas, Mylpf and Myl1 (Figure 4A,B), which are critical for maintaining muscle mass, driving myogenesis and regulating muscle contractility. These findings suggest that HGWD might preserve muscle structure and function in lung cancer cachexia through the modulation of metabolic and motor protein pathways.

**FIGURE 4:**
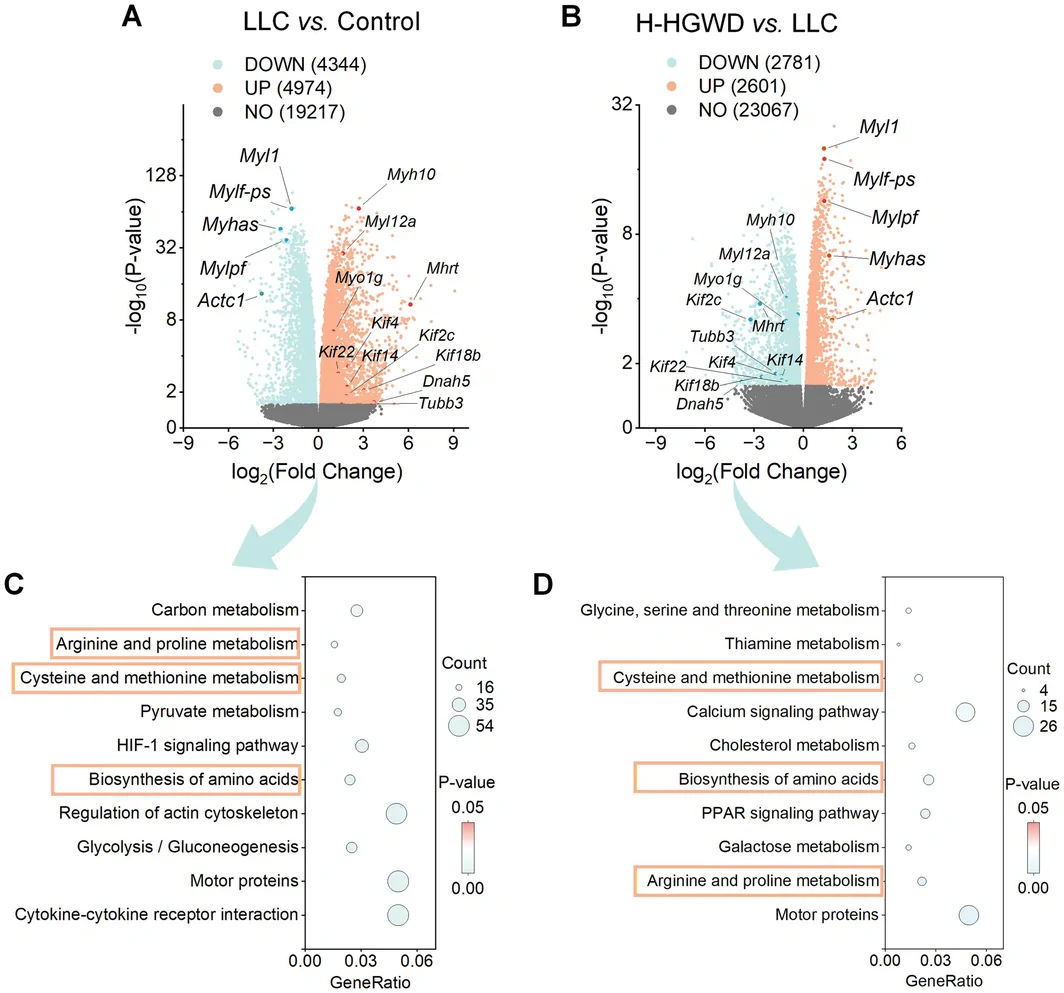
Impact of HGWD on transcriptomics of gastrocnemius muscle in cachectic mice. Differentially expressed genes (DEGs) between LLC vs. Control and H‐HGWD vs. LLC (A, B). KEGG pathway enrichment analysis is also provided (C, D).

### 3.4 Effects of HGWD on the Untargeted Metabolome of Cachectic Mice

Given our transcriptome findings that HGWD modulates multiple amino acid metabolism pathways—including ‘Arginine and proline metabolism’, ‘Biosynthesis of amino acids’ and ‘Cysteine and methionine metabolism’—we performed untargeted metabolomic profiling of gastrocnemius muscles from the Control LLC and H‐HGWD groups to clarify how HGWD regulates muscle metabolism to improve tumour cachexia. PCA revealed that samples within each group clustered tightly and showed distinct separation among groups, indicating that HGWD markedly altered the aberrant metabolite profile of cachectic gastrocnemius muscle (Figure 5A). Partial least squares discriminant analysis (PLS‐DA) further demonstrated clear separation of PLS‐DA score plots for LLC vs. Control and H‐HGWD vs. LLC, with R^2^Y values exceeding 0.9 and Q^2^ values below −0.2. These parameters confirm that the PLS‐DA models were robust, not overfitted and had strong explanatory power (Figure 5B–E). We identified differential metabolites using thresholds of FC > 1.5 or FC < 0.67, VIP > 1.0 and *p* < 0.05 and subjected these metabolites to KEGG pathway enrichment analysis. Differential metabolites from both LLC vs. Control and H‐HGWD vs. LLC comparisons were significantly enriched in ‘Biosynthesis of amino acids’ (Figure 5F,G). Close examination of amino acid biosynthesis–related metabolites showed that HGWD upregulated most branched‐chain and essential amino acids, including S‐adenosylmethionine (SAM), L‐arginine, L‐tryptophan, L‐ornithine, L‐leucine and spermidine (Figure 5H). These metabolites are well known to enhance protein synthesis, support myogenesis and alleviate sarcopenia and cachexia. Collectively, these results indicate that HGWD confers therapeutic benefits against lung cancer cachexia, potentially through rectifying amino acid metabolic dysregulation in the gastrocnemius muscle, reinstating metabolic homeostasis and sustaining muscle mass and contractile function.

**FIGURE 5:**
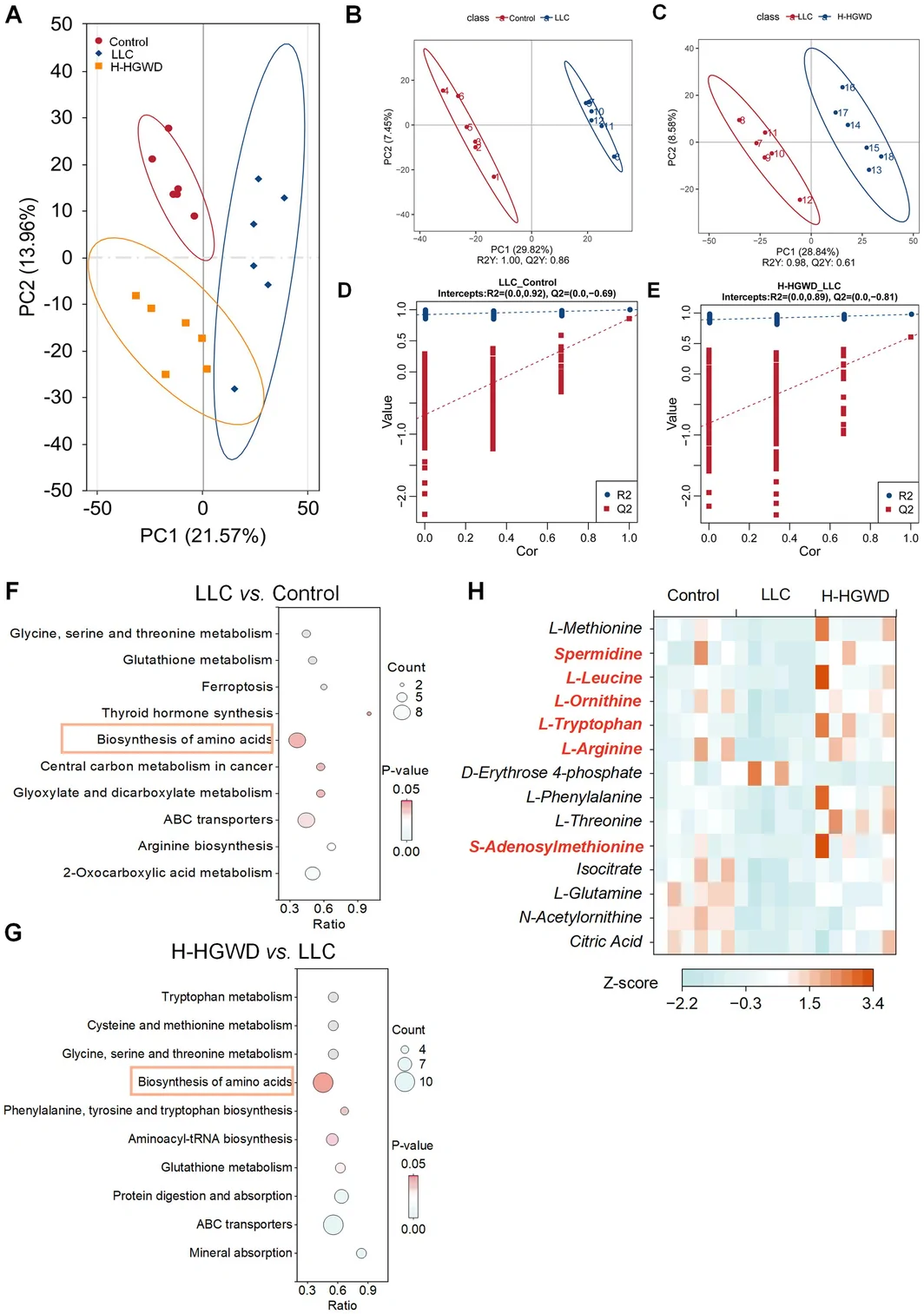
Impact of HGWD on untargeted metabolomics of gastrocnemius muscle in cachectic mice. (A) PCA analysis, (B–E) PLS‐DA analysis, (F, G) KEGG analysis of differential metabolites between LLC vs. Control and H‐HGWD vs. LLC, (H) and heatmap of expression of metabolites related to ‘Biosynthesis of amino acids’.

### 3.5 HGWD Improves Amino Acid Metabolism in Cachectic Mice

We next examined gene expression profiles associated with ‘Arginine and proline metabolism’, ‘Biosynthesis of amino acids’ and ‘Cysteine and methionine metabolism’. HGWD intervention markedly upregulated genes linked to cachexia improvement—*Amd1, Amd2, Smox* and *Ckm*—while downregulating *Gnmt* (Figure 6A), whose dysregulation is known to disrupt amino acid metabolism. Integrating these transcriptomic data with our untargeted metabolome findings, we propose that HGWD alleviates tumour cachexia by suppressing GNMT, increasing the SAM/SAH ratio and enhancing AMD1 and SMOX expression. These changes likely promote the conversion of SAM to spermidine, a key metabolite associated with muscle maintenance and metabolic homeostasis (Figure 6B). Consistent with this hypothesis, Western blot analysis confirmed that HGWD treatment increased the protein levels of AMD1 and SMOX while decreasing GNMT protein expression in the gastrocnemius muscle of cachectic mice (Figure 6C–F). These results provide initial mechanistic evidence supporting our speculation.

**FIGURE 6:**
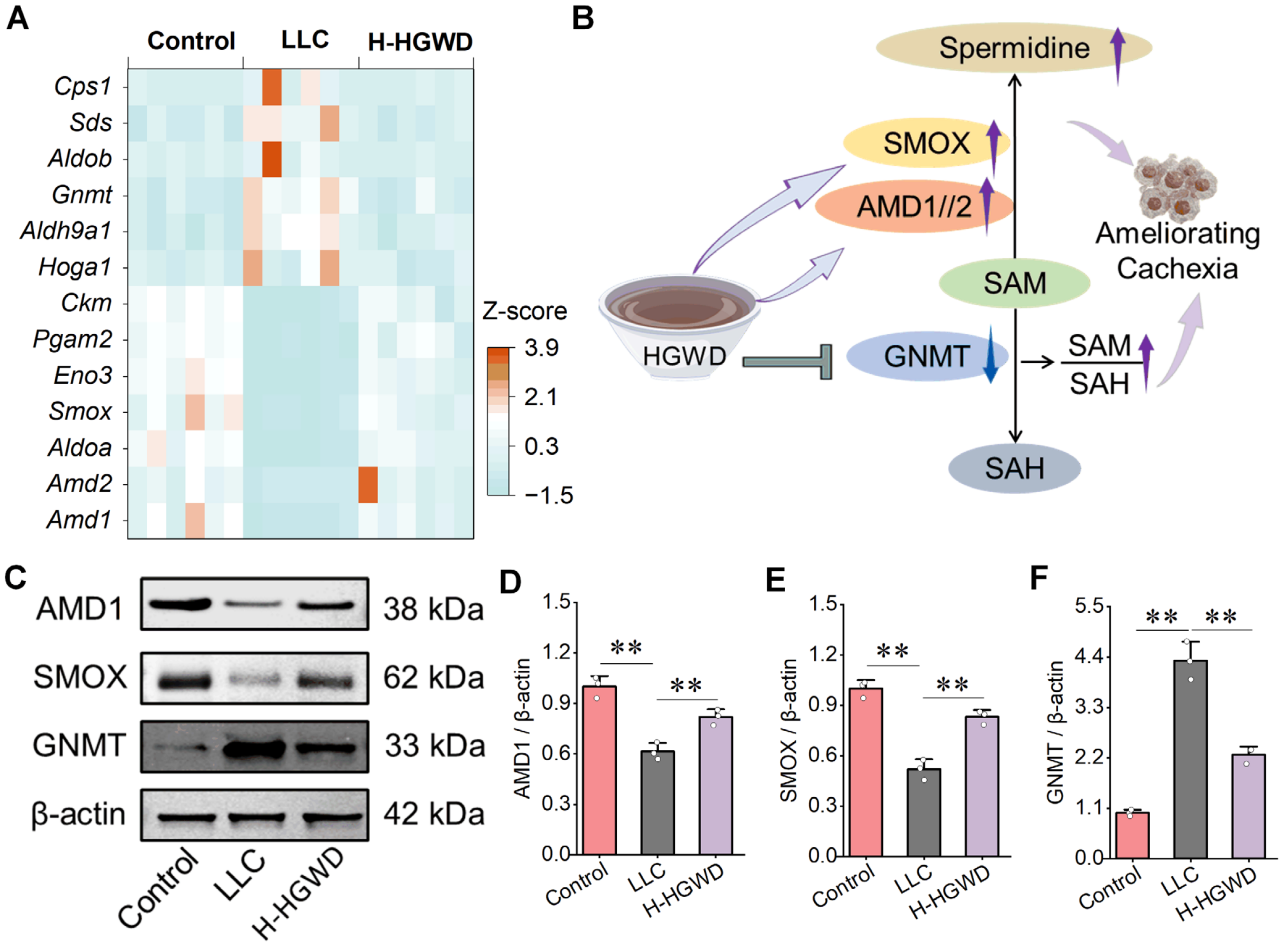
HGWD improves amino acid metabolism in gastrocnemius muscle of cachectic mice. (A) Heatmap of shared gene expression in ‘Arginine and proline metabolism’, ‘Biosynthesis of amino acids’ and ‘Cysteine and methionine metabolism’, (B) Schematic diagram of the proposed mechanism by which HGWD ameliorates cachexia. (C–F) HGWD upregulates protein expression of AMD1 and SMOX (C–E) and downregulates expression of GNMT (C, F) in gastrocnemius muscle of cachectic mice.

## 4 Discussion

Tumour cachexia is a multifactorial metabolic syndrome that significantly impairs patient outcomes. Epidemiological studies indicate that over 50% of cancer patients develop cachexia, which directly accounts for up to 20% of cancer‐related deaths [25]. Its hallmark features include anorexia, persistent weight loss and the degradation of skeletal muscle and adipose tissue, with profound muscle wasting being the most critical determinant of patient quality of life and survival [5]. Despite its clinical significance, no effective or specifically targeted therapies currently exist. The LLC‐induced tumour cachexia model in vivo is widely used to investigate the mechanisms of cachexia and to screen potential therapeutic interventions [18]. In this study, we assessed the effects of HGWD on muscle function and metabolism in cachectic mice. Muscle strength was evaluated using GST and WGT, while skeletal muscle mass and cross‐sectional area were measured. We also quantified proteins essential for myogenesis and examined metabolic and oxidative stress–related factors in skeletal muscle. HGWD treatment significantly improved muscle strength and mass, upregulated myogenesis‐related proteins and restored metabolic and oxidative stress markers in cachectic mice, demonstrating its protective effects against lung cancer–induced cachexia. Notably, HGWD also exhibited some tumour‐suppressive activity. In contrast to CDDP, which exacerbated cachexia despite its antitumor effects, HGWD simultaneously mitigated cachexia and inhibited tumour growth, underscoring its potential as a dual‐function therapeutic agent for lung cancer–associated cachexia. Noteworthily, increased food consumption in the HGWD‐treated group could contribute to the observed amelioration of cachexia; therefore, a pair‐feeding experiment would be necessary to definitively distinguish direct metabolic effects from nutritional improvements.

Transcriptomic analysis suggested that HGWD ameliorates tumour cachexia primarily by modulating amino acid metabolism. Under normal physiological conditions, homeostatic amino acid metabolism sustains muscle protein synthesis and functional stability [6]. In pathological states, disruption of amino acid metabolism induces muscle metabolic imbalance, impairs protein synthesis and compromises muscle function, thereby accelerating tumour cachexia progression [7]. Our analysis revealed significant enrichment and overlap of pathways including ‘Arginine and proline metabolism’, ‘Biosynthesis of amino acids’ and ‘Cysteine and methionine metabolism’, highlighting HGWD's regulatory impact on these key amino acid metabolic pathways. Dysregulation of arginine metabolism is known to inhibit protein synthesis and promote proteolysis, contributing directly to cachexia [26]. Similarly, interruption of methionine metabolism drives muscle atrophy in tumour cachexia, whereas deletion of key enzymes that block methionine metabolism can reverse this effect [7]. Supplementation with essential amino acids such as arginine and methionine has been shown to partially mitigate cachexia progression [7, 27]. Notably, HGWD upregulated numerous genes associated with ‘Motor proteins’, including Myhas, Mylpf and Myl1, which are critical for maintaining muscle mass, supporting myogenesis and preserving contractile function [28, 29, 30]. These findings provide mechanistic evidence that HGWD counteracts muscle atrophy in cachexia by restoring amino acid metabolic balance and enhancing the expression of proteins essential for muscle structure and function.

Building on our transcriptomic findings, we used untargeted metabolomics to examine how HGWD regulates amino acid metabolism in cachectic muscle. HGWD treatment significantly increased levels of branched‐chain and essential amino acids, including SAM, L‐arginine, L‐tryptophan, L‐ornithine, L‐leucine and spermidine. The beneficial role of arginine in mitigating cachexia has been well documented. Accelerated tryptophan catabolism contributes to sarcopenia progression and its inhibition represents an effective therapeutic strategy [31]. Similarly, ornithine [32, 33] and leucine [34] supplementation have been shown to improve muscle mass and function in cachectic conditions. Spermidine, produced from SAM through decarboxylation, plays a critical role in protecting against muscle ageing and maintaining muscle homeostasis [35].

Transcriptomic analysis of amino acid metabolism–related genes further revealed that HGWD enhanced the expression of key enzymes involved in the conversion of SAM to spermidine. Specifically, HGWD upregulated *Amd1, Amd2* and *Smox* while downregulating *Gnmt*. AMD1 and AMD2, members of the adenosylmethionine decarboxylase family, catalyse the conversion of SAM to decarboxylated SAM, providing propylamine groups for polyamine synthesis, including spermidine and serve as rate‐limiting enzymes in this pathway. Reduced expression of AMD1 disrupts polyamine metabolism, contributing to muscle atrophy [36]. SMOX is a key enzyme that converts decarboxylated SAM into spermidine and its expression positively correlates with muscle mass and function; elevated SMOX levels can alleviate sarcopenia progression [37]. GNMT catalyses the conversion of SAM to S‐adenosylhomocysteine (SAH). Dysregulated GNMT expression reduces the SAM/SAH ratio, a change closely linked to muscle ageing and the progression of cachexia [38]. In our study, HGWD not only restored levels of key branched‐chain amino acids but also normalized the expression of enzymes governing their metabolic pathways. These findings suggest that the alleviation of lung cancer–associated cachexia by HGWD is related to enhanced conversion of SAM to spermidine and restoration of amino acid metabolic homeostasis.

However, several issues need to be mentioned. First, although preliminary metabolomic analysis suggested that the muscle‐protective effect of HGWD may be related to amino acid metabolism, we cannot exclude the possibility that this effect is secondary to reduced tumour burden rather than a direct metabolic action within muscle tissue. Future studies will employ an in vitro C2C12 myotube atrophy model induced by tumour‐derived catabolic factors, such as TNF‐α or proteolysis‐inducing factor (PIF), followed by treatment with HGWD‐containing serum. Second, our findings are currently correlational and lack direct causal evidence. Future investigations will address this limitation by employing an LCM‐induced in vitro atrophy model treated with HGWD‐containing serum, combined with mechanistic studies involving siRNA‐mediated knockdown of AMD1 or SMOX and the application of specific enzyme inhibitors.

## 5 Conclusion

HGWD effectively mitigates cachexia in lung cancer–bearing mice by increasing muscle strength and mass and by improving muscle metabolism and oxidative stress. Mechanistically, HGWD appears to promote the SAM‐to‐spermidine conversion, correct amino acid metabolic disturbances and exert a therapeutic effect on lung cancer–induced cachexia (Figure 7). This study elucidates both the role and mechanism of HGWD in tumour cachexia, providing a foundation for its broader clinical application. Nevertheless, several questions remain. The primary bioactive components of HGWD responsible for cachexia improvement and the precise molecular targets mediating its effects on amino acid metabolism are still unknown. Future studies integrating metabolomics, transcriptomics and proteomics, combined with predictive modelling using deep learning and validated through techniques such as surface plasmon resonance (SPR) and drug affinity responsive target stability (DARTS), are expected to uncover the detailed mechanisms by which HGWD ameliorates cachexia.

**FIGURE 7:**
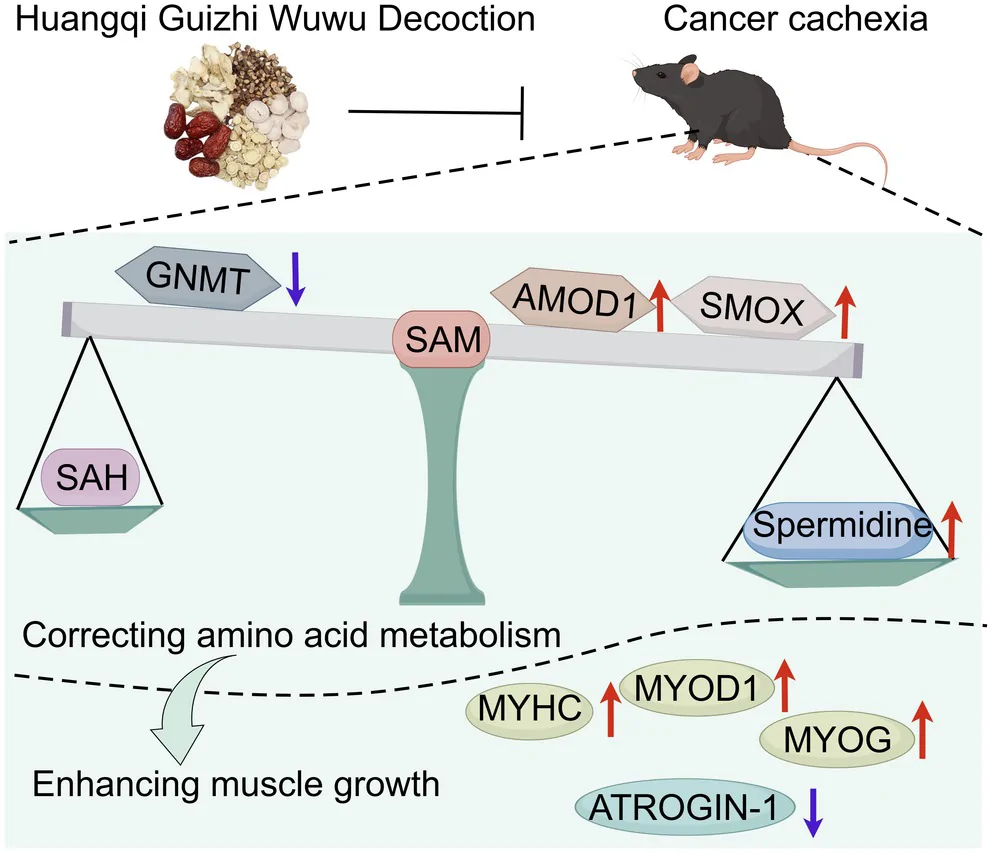
Schematic diagram of the mechanism by which HGWD ameliorates cachexia. HGWD promotes conversion of SAM to spermidine, corrects amino acid metabolism disorders and exerts therapeutic effects on lung cancer cachexia.

## Author Contributions

**Yingjia Zhou:** formal analysis, investigation, writing – original draft. **Shaoli Zhao:** formal analysis, investigation, writing – original draft. **Hongmei Shen:** methodology. **Jia Wu:** data curation. **Mei Li:** supervision. **Shan Liu:** validation. **Jian Yang:** visualization. **Xiaowei Jin:** writing – review and editing. **Fan Li:** writing – review and editing. **Huantian Cui:** conceptualization.

## Funding

This work was supported by the Yunnan Provincial Famous Veteran Traditional Chinese Medicine Experts Inheritance Studio Construction Project (Famous TCM Expert of Guangdong Province, Professor Lin Lizhu) (Grant No. CZ0194), the Yunnan Provincial Pilot Project for Clinical Collaboration of Chinese and Western Medicine in Major Difficult Diseases (Lung Cancer) (Grant No. CZ0193), the First‐Class Discipline Team of Kunming Medical University (Pharmaceutical Policy Research and Practice) (Grant No. 2024XKTDPY21) and the National Construction Project for the ‘Flagship’ Departments of Integrated Chinese and Western Medicine Collaboration.

## Conflicts of Interest

The authors declare no conflicts of interest.

## Supporting information


**Data S1:** Supporting Information.
**Figure S1:** Impact of HGWD on tumour growth and consumption in cachectic mice.
**Table S1:** Detailed information of main compounds in HGWD.
**Table S2:** Detailed information of HGWD prototype compounds in serum.

## Data Availability

The transcriptomics data has been deposited in the Genome Sequence Archive (GSA) under accession number CRA048164. The metabolomics data has been deposited to MetaboLights [39] repository with the study identifier MTBLS15264.
