# Supplementary material for: Huangqi Guizhi Wuwu Decoction Ameliorates Cachexia in Lung Cancer Mice Through Regulating Amino Acid Metabolism: Insights From Transcriptomics, Metabolomics and Experimental Validation
Source: J Cell Mol Med. 2026 Sep 27;30(19):e71387. doi: 10.1111/jcmm.71387 (PMC13616822; doi:10.1111/jcmm.71387)
Supplement: Supplementary file 1 [file JCMM-30-e71387-s001.docx]

**Supplementary material**

**1 Reagents**

Cisplatin (HY-17394) was purchased from Shanghai Medchemexpress Bio-Technology Co., Ltd. (Shanghai, China). HE ([D006-1-1](http://www.njjcbio.com/products.asp?id=476)) staining assay kit was purchased from Nanjing Jiancheng Biological Engineering Institute (Nanjing, China). Muscle Glycogen Assay Kit (A043-1-1), SOD (A001-3-2), GSH-PX (A005-1-1), MDA (A003-1-2) were purchased from Nanjing Jiancheng Biological Engineering Institute (Nanjing, China). Lactic Acid (LA) Content Detection Kit (BL868B) was purchased from Biosharp Life Sciences. MYHC (20140-1-AP), MYOD1 (18943-1-AP), MYOG (26762-1-AP) and ATROGIN-1 (67172-1-Ig) were purchased from Proteintech (Wuhan, China). GNMT (A03562-2), AMD1 (A06146) and SMOX (A05519-1) were purchased from BOSTER Biological Technology Co., Ltd.

**2 The decoction method of Huangqi Guiizi Wuwu Decoction**

Huangqi Guizhi Wuwu Decoction (HGWD): Astragali Radix (huangqi) 9 g. Cinnamomi Ramulus (guizhi) 9 g, Paeoniae Radix (shaoyao) 9 g, Zingiberis Rhizoma Recens (shengjiang) 18 g, Jujubae Fructus (dazao) 12 pieces (≈ 36 g).

Decoction procedure: Place the five ingredients in a vessel at the above ratio, add de-ionized water, and soak at room temperature for 30 min, stirring twice to ensure full hydration. Bring rapidly to a boil, then maintain a gentle simmer for 30 min. While still hot, strain the liquid through gauze. Return the dregs to the pot, add fresh de-ionized water, bring to a boil again, and simmer gently for a further 20 min; filter in the same way. Combine the two extracts and reduce to the desired volume either under reduced pressure or by slow evaporation over a low flame.

**3 Qualitative analysis of Huangqi Guizhi Wuwu Decoction**

**3.1 Instruments**

Agilent 1290 Infinity II Ultra-Performance Liquid Chromatography (UPLC) system (Agilent, USA); high-resolution mass spectrometer (Q-TOF G6545, Agilent, USA); chromatographic column: Agilent EclipsePlus C18 RRHD (1.8 μm, 2.1 mm × 100 mm).

**3.2 HGWD Extraction and serum preparation containing HGWD**

One gram of HGWD was weighed and extracted with 10 mL of methanol by ultrasonication for 30 min. The mixture was then centrifuged at 3000 rpm for 10 min. The supernatant was filtered through a 0.22 μm microporous membrane, and the filtrate was transferred into a UPLC vial for analysis.

Mice were administered HGWD by intragastric gavage at a high dose for 3 consecutive days. Plasma was collected at 1 h and 2 h after the last administration and mixed uniformly. Methanol was added to the plasma at a volume ratio of 1:3 (plasma:methanol), vortexed for 30 s, and allowed to stand at 4 °C for 20 min. The mixture was then centrifuged at 12,000 × g for 15 min, and the supernatant was collected and vacuum-dried. The residue was reconstituted in 100 μL methanol, centrifuged at 12,000 × g for 10 min, and the supernatant was collected. This supernatant was again vacuum-dried, reconstituted in 100 μL methanol, and centrifuged at 12,000 × g for 10 min. The final supernatant was transferred to an autosampler vial for subsequent instrumental analysis.

**3.3 Chromatographic Conditions**

Chromatographic separation was performed on an Agilent 1290 Infinity II UPLC system equipped with an Agilent EclipsePlus C18 column (2.1 mm × 100 mm, 1.8 μm).

In positive ion mode, the mobile phase consisted of (A) 0.1% formic acid in water and (B) 0.1% formic acid in acetonitrile, using a gradient elution.

In negative ion mode, the mobile phase consisted of (A) 0.01% formic acid in water and (B) 0.01% formic acid in acetonitrile, also under gradient elution.

The gradient program was as follows:

0–5.0 min, 2% B;

5.0–20.0 min, 2% → 100% B;

20.0–25.0 min, 100% B;

25.0–30.0 min, 100% → 2% B;

30.0–33.0 min, 2% B.

Column temperature: 40 °C; flow rate: 0.4 mL/min; injection volume: 5 μL.

**3.4 Mass Spectrometric Conditions**

An electrospray ionization (ESI) source was used for data acquisition in both positive and negative ion modes. Drying gas temperature: 320 °C; drying gas flow: 8 L·min⁻¹; Sheath gas temperature: 350 °C; sheath gas flow: 11 L·min⁻¹; Nebulizer pressure: 35 psi; Nozzle voltage: 3.5 kV; Capillary voltage: 1000 V; Mass scan range: *m/z* 100–1000; Collision energy: 10, 20, and 40 eV (stepped); High-purity nitrogen was used as the nebulizer gas, drying gas, and sheath gas.

**3.5 Data analysis**

The MS data was processed by Agilent MassHunter Workstation Qualitative Analysis 10.0 (Agilent, USA) with the steps of raw data introduction, peak extraction and adduct deconvolution. The identification was finally determined by consideration of retention time error of reference substance, mass error of mother ion, match degree of daughter ions, isotope distribution and peak area after searching the reference substance database (Agilent- Natural Standard NPs PCDL, NatureStandard Shanghai Standard Technology Co., Ltd.) and theoretical database constructed by literature and public databases.

**3.6 Results**

Base Peak Chromatogram (BPC) of HGWD extraction and serum samples can be found in Figure 1. Detailed information of the main compounds in HGWD and its prototype compounds in plasma can be found in Table S1 and S2.

**4** **Supplementary figures**

**Figure S1 Impact of HGWD on tumor growth** **and consumption in cachectic mice.**

HGWD reduces tumor volume (A) and tumor weight (B) in cachectic mice, increases tumor inhibition rates (C), and improves intake of food (D) and water (E). ns: not significant, **P* < 0.05, ***P* < 0.01.

**5 Supplementary tables**

**Table S1.** Detailed information of main compounds in HGWD.

| Identification | Formula | Adduct | Theoretical m/z | Mass Error (ppm) | RT (min) |
| --- | --- | --- | --- | --- | --- |
| 6-Gingerol | C₁₇H₂₆O₄ | (M-H)⁻ | 293.1748 | +1.79 | 13.48 |
| Calycosin | C₁₆H₁₂O₅ | (M-H)⁻ | 284.0678 | +1.85 | 10.46 |
| Astragaloside IV | C₄₁H₆₈O₁₄ | (M+CH₃COO)⁻[-H₂O] | 825.461 | +2.50 | 12.71 |
| Paeoniflorin | C₂₃H₂₈O₁₁ | (M+HCOO)⁻ | 525.1592 | +1.04 | 8.51 |
| Ethyl-p-methoxycinnamate | C₁₂H₁₄O₃ | (M+2HCOO)⁻²[-H₂O] | 206.0949 | +4.16 | 7.36 |
| Oleanolic acid | C₃₀H₄₈O₃ | M+4 | 456.3614 | -4.82 | 20.84 |
| Acetylacteoside | C₃₁H₃₈O₁₆ | (M+Na)⁺ | 689.2046 | -0.97 | 1.03 |

**Table S2.** Detailed information of HGWD prototype compounds in serum.

| Identification | Formula | Adduct | Theoretical m/z | Mass Error (ppm) | RT (min) |
| --- | --- | --- | --- | --- | --- |
| 6-Gingerol | C₁₇H₂₆O₄ | (M-H)⁻ | 293.1748 | +3.50 | 13.48 |
| Calycosin | C₁₆H₁₂O₅ | (M-H)⁻ | 284.0678 | +4.38 | 10.41 |
| Astragaloside IV | C₄₁H₆₈O₁₄ | (M+CH₃COO)⁻[-H₂O] | 825.461 | +2.13 | 12.70 |
| Paeoniflorin | C₂₃H₂₈O₁₁ | (M+HCOO)⁻ | 525.1592 | +1.42 | 8.69 |
| Ethyl-p-methoxycinnamate | C₁₂H₁₄O₃ | (M+2HCOO)⁻²[-H₂O] | 206.0949 | +0.56 | 7.56 |
| Oleanolic acid | C₃₀H₄₈O₃ | M+4 | 456.3614 | -2.19 | 20.84 |
| Acetylacteoside | C₃₁H₃₈O₁₆ | (M+Na)⁺ | 689.2046 | -0.54 | 1.02 |
